# Scramblases as Regulators of Autophagy and Lipid Homeostasis: Implications for NAFLD

**DOI:** 10.1080/27694127.2022.2055724

**Published:** 2022-04-07

**Authors:** Allen Chen, Wen-Xing Ding, Hong-Min Ni

**Affiliations:** a Department of Pharmacology, Toxicology and Therapeutics, The University of Kansas Medical Center, Kansas City, KS 66160, USA; b Department of Internal Medicine, The University of Kansas Medical Center, Kansas City, KS 66160, USA

**Keywords:** autophagy, NASH, TMEM41B, VLDL, VMP1

## Abstract

Equilibration of phospholipids between the two monolayers of the lipid bilayer of cellular membranes is mediated by scramblases acting as phospholipid shuttling proteins that are critical for cellular function, particularly during inter-organelle contact. Recent work has identified several protein scramblases, including TMEM41B, VMP1 and ATG9 that are critical in autophagy. More recently, ATG9, TMEM41B, and VMP1 have also been discovered to be important regulators of cellular lipid homeostasis. In vivo mouse models involving ablation of TMEM41B in liver have shown that knockout of these proteins can lead to rapid development of non-alcoholic steatohepatitis (NASH) and systemic dyslipidemia, though this has not been explored yet with ATG9. The resulting phenotype is likely due to the combined effects of a severe lipid secretion defect caused by stalled neutral lipids export from the endoplasmic reticulum (ER) membrane bilayer coupled with increased lipogenesis. Here we briefly discuss recent exciting findings on the topic of scramblases in autophagy, their relevance to human non-alcoholic fatty liver disease (NAFLD)/NASH, as well as future directions in this research.

## Introduction

Membrane dynamics is required for organelle formation and transport and thus are integral to normal cell functionality. Membranes are needed to establish intracellular compartments/organelles, separating them from the cytosol so that these organelles can perform their complex and unique functions. Macroautophagy (hereafter referred to as autophagy) is a highly conserved lysosome-mediated degradation pathway allowing for adaptive survival in response to stresses such as starvation ^1^. A distinguishing feature of autophagy is dynamic membrane remodeling to form double-membraned autophagosomes. Although the exact membrane sources for autophagosome formations are not yet completely known, it has been widely accepted that autophagosomes originate from isolation membranes (IMs) (also called phagophores) near specific double-FYVE-containing protein 1 (DFCP1)-positive areas on the endoplasmic reticulum (ER). The IM is then nucleated, elongated, and closed to form double membrane autophagosomes, which eventually fuse with lysosomes to form autolysosomes, resulting in the autophagosome contents and inner membrane being degraded by lysosome hydrolases ^1–3^. While many autophagy related (ATG) proteins have been identified and extensively studied in regulating autophagy process, little is known about the role of autophagosomal lipids and lipid compositions on autophagosome membrane shaping and remodeling during autophagy.

**Figure 1.**
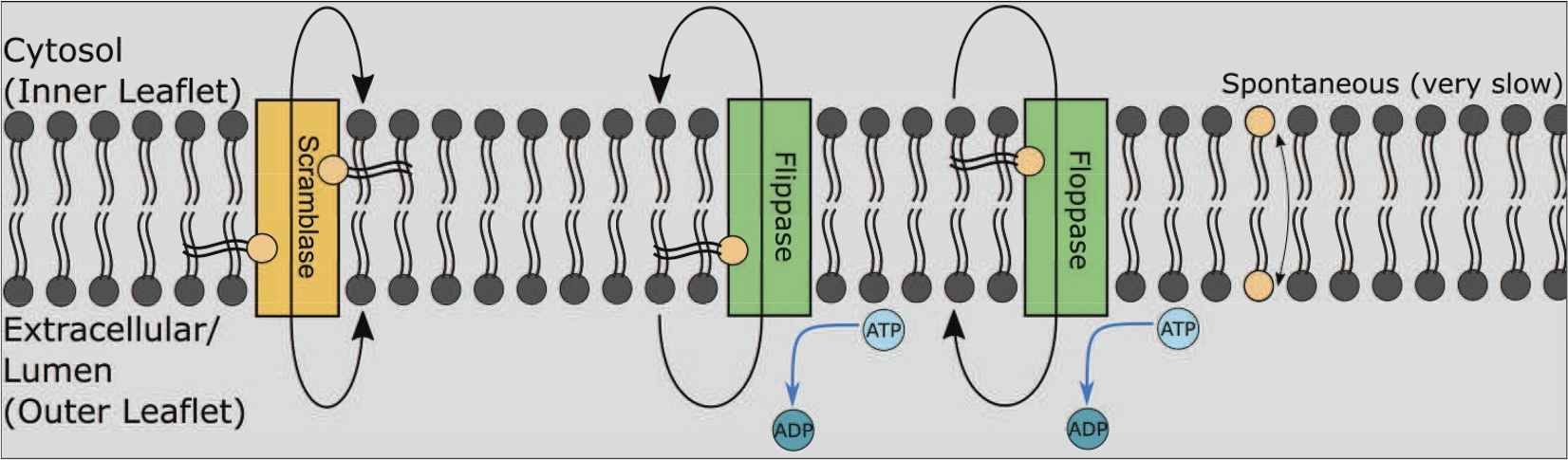
The three types of phospholipid translocases.

One seldom-discussed type of membrane protein important in a diverse set of processes involving cellular membrane remodeling and autophagy is the phospholipid scramblase, which has only recently become a hot topic of research. Phospholipid scramblases are primarily known to be involved in autophagosome and lipid droplet (LD) formation, largely by balancing the number of phospholipids between the two leaflets of phospholipid bilayers ^4^. In contrast to flippases and floppases, which also catalyze phospholipid transport between apposed bilayers, scramblases are both ATP-independent and bidirectional, and thus may be considered as transporters involved in facilitated diffusion of phospholipids, whereas flippases and floppases are more analogous to proteins involved in active transport (Figure 1). Among the scramblases implicated in autophagy are ATG9 localized to autophagic vesicles and TMEM41B (Transmembrane Protein 41B) and VMP1 (Vacuole Membrane Protein 1) localized to the ER membrane ^5–8^. While all three proteins are considered scramblases, they are not redundant, and loss of any of these proteins can lead to dramatic loss of autophagy and normal cellular functioning.

Non-alcoholic fatty liver disease (NAFLD) is one of the leading causes of chronic liver disease in the United States (U.S.), with an estimated 24% of U.S. adults having some form of the condition ^9^. NAFLD is characterized by greater than five percent hepatic steatosis without evidence of other causes of liver disease, such as viral hepatitis or drugs and medications. When NAFLD is present with inflammation, the condition is given the name non-alcoholic steatohepatitis (NASH). Chronic liver diseases, including NASH, can eventually progress to more severe conditions including cirrhosis and hepatocellular carcinoma (HCC) ^1^. Health conditions such as obesity, metabolic syndrome, and type 2 diabetes mellitus (T2DM) can increase the likelihood of developing NAFLD ^9^. High prevalence of this disease coupled with the rising incidence of diseases such as obesity and T2DM indicate that NAFLD and sequelae may soon become a significant burden to the U.S. population. Currently, there are no pharmacological treatments available for NAFLD or NASH, so weight loss and dietary changes, which are effective but rarely sustainable, are usually prescribed to target associated risk factors such as obesity and T2DM ^10,11^.

Hepatic accumulation of lipids is generally thought to occur because of breakdown in the balance between lipid accumulation and elimination due to dysfunctional control over the following four mechanisms: hepatic lipid uptake, de novo lipogenesis, fatty acid oxidation, and lipid secretion and excretion. Hepatic lipid uptake, largely mediated by fatty acid transporters including fatty acid transport proteins (FATPs), cluster of differentiation 36 (CD36), and caveolins, and de novo lipogenesis, in which new fatty acids are synthesized from acetyl coenzyme A (acetyl-CoA), are mechanisms by which the liver accumulates lipids and thus upregulation of these two pathways may contribute to NAFLD ^12^. In contrast, fatty acid oxidation, ATP generation from fatty acids mediated by peroxisome proliferator-activated receptor α (PPARα), and lipid excretion from the liver following fatty acid packaging into very-low-density lipoprotein (VLDL) particles, are the only mechanisms by which the liver eliminates lipids and thus counteract hepatic steatosis ^12, 13^. Unfortunately, oxidation of fatty acids, particularly in lipid overload due to NAFLD, can result in oxidative damage to the liver. Thus, for liver-sparing treatment of NAFLD, enhanced lipid export, rather than lipid metabolism, may be the preferred goal. Scramblase proteins, particularly TMEM41B and VMP1, have recently been shown to be critical in mediating lipoprotein secretion from intestine of zebrafish, cultured hepatoma cells and mouse livers, with loss of these proteins being associated with massive hepatic steatosis and near complete depletion of serum lipid levels in mouse models ^14, 15^. In this short commentary, we review recent progress in our understanding of lipid scramblases in regulating autophagy and lipid metabolism as well as the implications of these findings in NAFLD/NASH.

## Structure and Function of Scramblase Proteins

**Figure 2.**
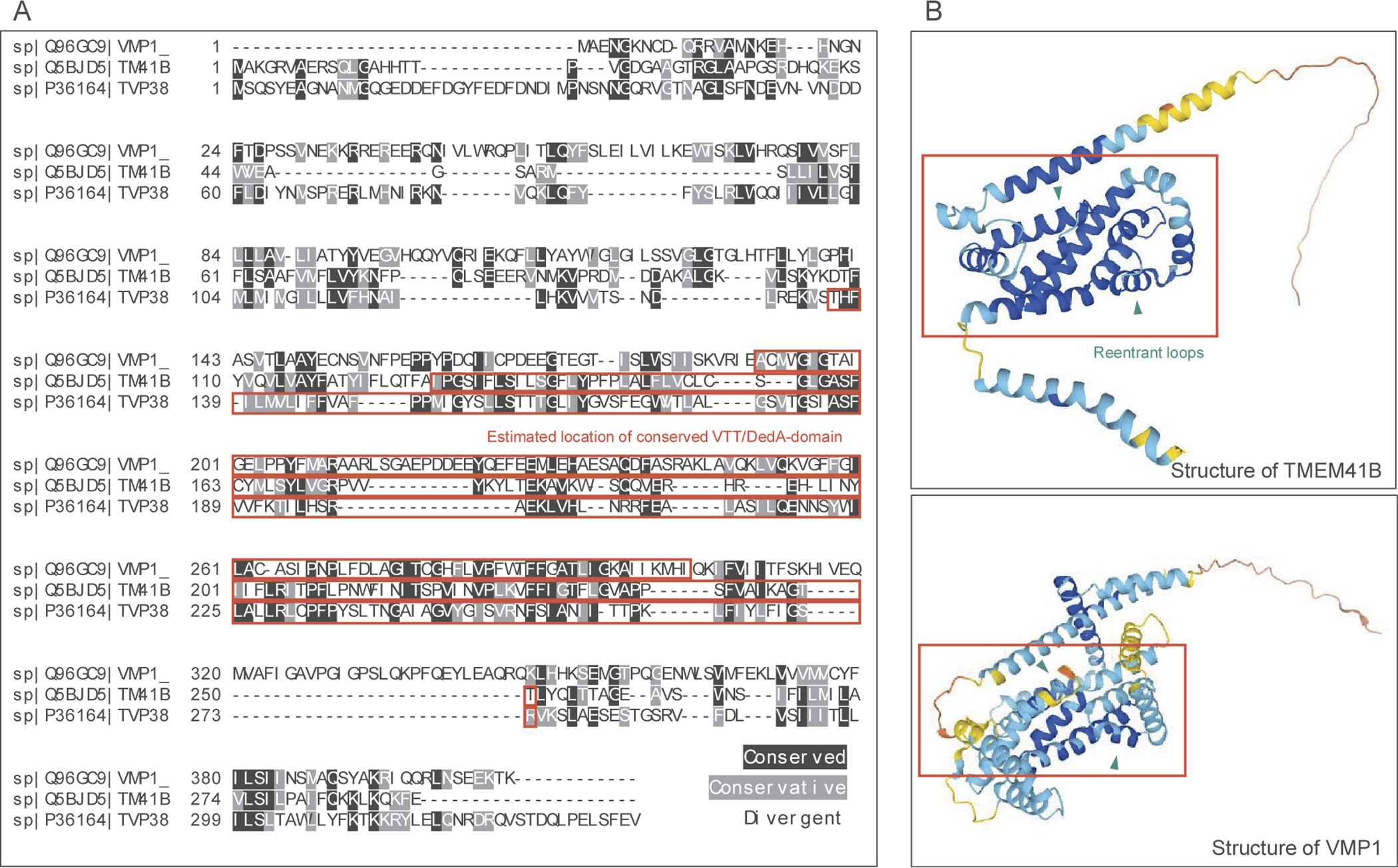
Sequence alignment and Structural Comparison of HsTMEM41B, HsVMP1.

TMEM41B and VMP1 are ER-resident membrane proteins that share an evolutionarily conserved domain originally termed the SNARE-associated domain. However, due to the dearth of evidence linking this domain to SNARE interaction, the conserved region was renamed the VTT (VMP1, TMEM41B and Tvp38) domain before being found to be homologous to regions in a host of other bacterial DedA family proteins and thus inducted into the DedA superfamily ^4, 8, 16^. A sequence alignment of VMP1, TMEM41B, and Tlg2p-vesicle protein 38 (Tvp38) illustrates the homology between the conserved VTT regions in each of the proteins (Figure 2A). Computer-generated models of VMP1 and TMEM41B, which, as opposed to Tvp38, are found in mammals including humans, illustrate the alpha helical structure common in many transmembrane proteins. Although having highly conserved sequences would imply that these proteins have critical roles in the cell, the functions of these proteins have only been sparsely studied before recently, largely in a limited number of disease models.

In recent publications, the protein structure of the DedA/VTT domain was predicted using the protein structure predicting software trRosetta as consisting of four transmembrane helixes along with two reentrant loops, helical domains that only extend part way through the lipid membrane before turning and exiting from the same side ^8, 17^. Further experiments were performed to confirm the structural model using a Substituted Cysteine Accessibility Method (SCAM) which allowed for identification of ER cytosolic-versus-lumen facing regions of the protein, confirming the four transmembrane helix and two reentrant loop structure of TMEM41B. Analysis of other protein structures revealed that dual reentrant loop-containing structures were found to be common in transporters such as aquaporins and ion transporters, where the reentrant loops were directly involved in substrate binding ^8^. In protein structures generated through AlphaFold, the dual-reentrant loops as well as the four transmembrane helixes as part of the DedA domain could be appreciated in high confidence prediction regions of HsTMEM41B, though low confidence predictions within the DedA domain of HsVMP1 model makes interpretation more difficult (Figure 2B). Okawa et al. also expressed some reservations with models of VMP1 due to a smaller number of homologous sequences than in TMEM41B and other VTT/DedA-domain proteins ^8^.

Recently, several groups have simultaneously reported that TMEM41B and VMP1 act as scramblases ^14, 18, 19^. Structural modelling on homologous proteins in prokaryotes as well as consideration of TMEM41B’s role in the ER-Golgi shuttle resulted in the hypothesis that TMEM41B is a lipid scramblase, responsible for bidirectional ATP-independent phospholipid flipping, equilibrating lipids between the inner and outer leaflets of lipid membrane bilayers. Phospholipid scramblase activity of TMEM41B and VMP1 was experimentally shown using a dithionite assay, an elegant assay in which fluorescence-tagged liposomes were treated with dithionite, a reducing agent unable to pass through the liposome membrane, leading to a 50% decline in fluorescence in control conditions and a 90% reduction in fluorescence when recombinant TMEM41B or VMP1 were added to the liposomes ^14, 18, 19^.

In addition to TMEM41B and VMP1, ATG9, previously the sole transmembrane autophagy-related protein, was also found to have lipid scramblase activity and to colocalize with ATG2 to regulate autophagosome membrane expansion and autophagy activity ^19–21^. Scramblase activity of ATG9 was also confirmed using a dithionite fluorescence assay ^2^. Interestingly, ablation of TMEM41B or VMP1, which are ER-resident proteins, but not ATG9, which exists on pre-autophagosome ATG9 vesicles, has been shown to result in lipid secretion defect ^14–16, 22^, suggesting that the locations of different scramblases may account for their distinctive roles aside from their role in regulating autophagy. Structural analysis of ATG9A using Cryo-EM revealed that this protein contained several structures conserved between human and yeast homologs: 1) four transmembrane helices with two helixes within the cytoplasmic leaflet, 2) a domain-swapped homotrimer architecture, in which helixes from one monomer interact with those of another, and 3) two unique pores, one transmembrane and the other lateral to the cytoplasmic leaflet of the membrane ^21, 23, 24^. The four transmembrane with two non-penetrating helix structure is reminiscent of the structures of TMEM41B and VMP1 and may be relevant to the scramblase activity of these proteins, though the transmembrane pore would be a more likely site of interleaflet movement in ATG9A and was confirmed to be vital for autophagy and autophagosome expansion in yeast and mammalian cells ^2, 21, 23^.

## Role of Scramblases in Autophagy

TMEM41B and VMP1 have been found to play critical roles in regulating autophagosome expansion and closure as well as in lipoprotein biogenesis, cholesterol distribution and lipid droplet (LD) homeostasis ^14–16, 18, 22^. TMEM41B was also recently found to be critical in mediating flavivirus and coronavirus infection, though its role in autophagy may not be needed for viral infection ^25, 26^. In contrast to ER-resident VMP1 and TMEM41B, ATG9A is specific to eponymous ATG9 vesicles which derive from the Golgi apparatus in a process requiring ATG23 and ATG27 in yeast and was thought to be the sole membrane source during early autophagosome formation ^5, 23^. ATG9 is now proposed to function in an ER-associated autophagosome membrane expansion process along with ATG2. In this process, ATG2 appears to act as a component in the ATG18-ATG2 complex which tethers the isolation membrane to the ER and transports lipids from the cytoplasmic leaflet of the ER to that of the isolation membrane ^27^. The scramblase ATG9 then shuttles these new lipids into the luminal leaflet. Following incorporation of the autophagosome with the lysosome, ATG9A is not degraded, but recycled back into ATG9A vesicles, a process that was recently found to be mediated by phosphatidylinositol 3-phosphate (PI3P)-binding protein sorting nexin 4 (SNX4) ^28^. Loss of control mechanisms such as SNX4 resulted in ATG9A accumulation on endolysosomes due to dysfunctional recycling and autophagy inhibition, illustrating the importance of ATG9 maintenance and lipid equilibration in such cellular processes. Ultimately, loss of ATG9A was shown to be associated with decreased autophagosome size, possibly due to a defect in bilayer structure resulting from phospholipid imbalance.

In contrast to ATG9, TMEM41B and VMP1 operate at exit sites along the ER as ER-resident scramblases. Although TMEM41B is known to be an important regulator of autophagy, TMEM41B depletion resulting in a marked stall in p62 clearance, the mechanism by which autophagy defect occurs in TMEM41B KO cells is still being elucidated ^22^. What has been found is that TMEM41B depletion is associated with an inability to form WD repeat domain, phosphoinositide interacting 2 (WIPI2)- and DFCP1-negative, mature autophagosomes. WIPI2 and DFCP1 are both ER-localized phosphatidylinositol 3-phosphate (PI(3)P) effector proteins recruited to the growing isolation membrane and are important for initiating autophagy. While DFCP1 is found on the ER and is associated with formation of omegasomes, ER-localized structures thought to act as platforms for autophagosome formation, WIPI2 has been shown to be involved in LC3 lipidation, forming LC3-II found on autophagosome membranes^29, 3^. Thus, TMEM41B depletion appears to inhibit autophagy at autophagosome closure and release from the ER, resulting in an accumulation of isolation membrane structures. TMEM41B depletion ultimately resulted in an accumulation of IMs, either indicating that they could not be cleared due to a defect in autophagy or due to an increase in production of isolation membranes due to defective maturation. Recent research, including the discovery that ATG9, VMP1 and TMEM41B forms a phospholipid transfer complex involving ATG2, provides evidence for VMP1, TMEM41B and ATG9 acting as complementary proteins on opposite ends of a phospholipid shuttle machinery involving ATG2 ^30, 31^, ensuring that phospholipids are transferred to expanding autophagosomes without disrupting autophagosome or ER ultrastructure.

**Figure 3.**
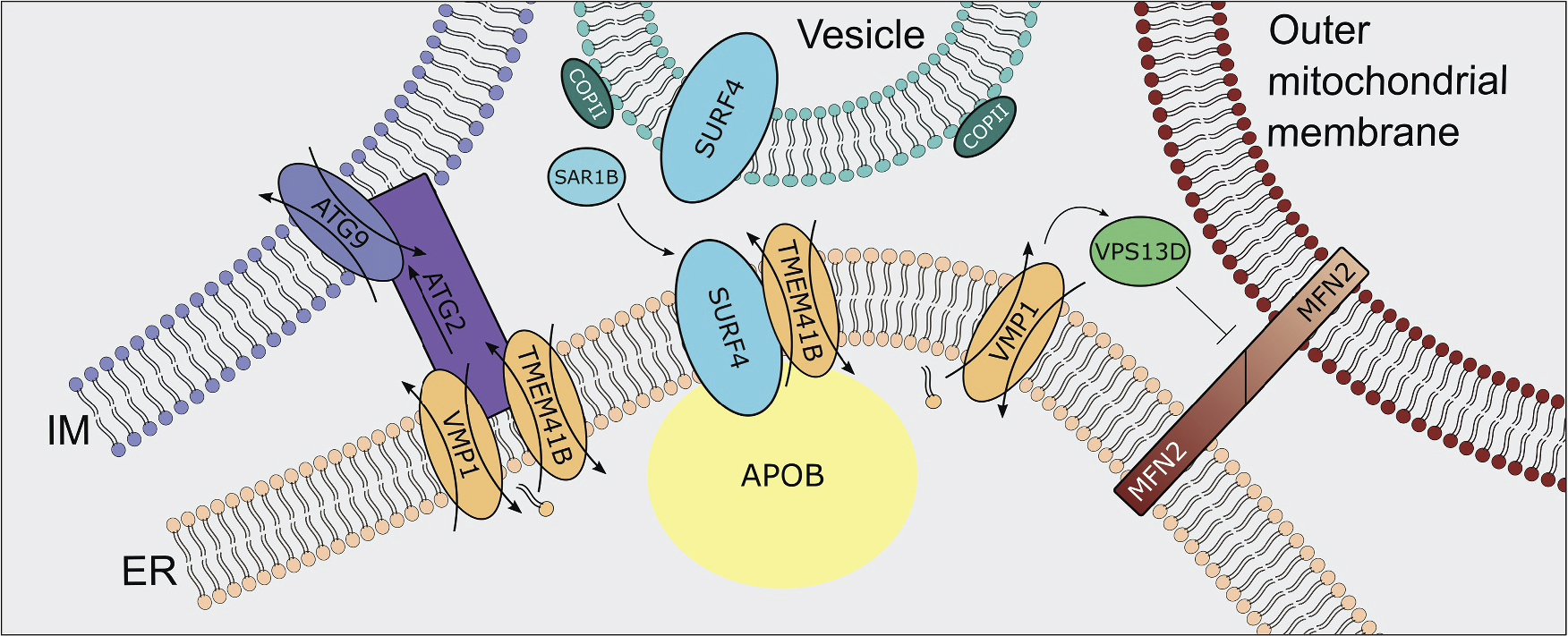
TMEM41B and VMP1 are major proteins at several ER-Organelle Contact Sites.

In addition to ATG2, an inter-organelle lipid transfer and anchoring protein involved in autophagosome expansion that interacts with TMEM41B, another protein that forms complexes with DedA domain scramblases is vacuolar protein sorting 13 homolog D (VPS13D), which was found to interact with VMP1 at mitochondria-ER contact sites. VPS13D is one of a family of proteins, some of which, including VPS13A and C, having been found to have roles in organelle contact and lipid transport ^32^. VPS13D, in contrast, is the only member of the family to contain a ubiquitin-binding domain and is thus involved in mitochondria clearance. Recent research using TEM analysis on ER-mitochondria contact has shown that loss of either VPS13D or VMP1 results in increased mitochondrial-ER contact as well as aberrant spherical mitochondria and inhibition of mitophagy in intestinal cells of *Drosophila* ^33^. Furthermore, combined knockdown of VMP1 and VPS13D did not result in enhanced mitophagy suppression phenotype compared to single knockdown of VMP1 or VPS13D, providing evidence for VMP1-VPS13D interaction in a single pathway ^33^. Mitochondrial-associated regulatory factor (marf)/mitofusin 2 (MFN2) overexpression in *Drosophila* and human cells was shown to result in a similar mitochondrial phenotype as VMP1/VPS13D loss, with knockdown of MFN2 remediating the effects of VMP1/VPS13D loss ^33^. Interestingly, here, interaction of VMP1 and VPS13D appears to inhibit inter-organelle contact, with VPS13D somehow interfering with the function of MFN2 in acting as an ER-mitochondria anchor. Although ATG2 and VPS13D may be implicated in lipid transfer processes and interact with scramblases, they appear to have opposed functions, with ATG2 acting as an anchor while VPS13D inhibits inter-organelle interaction (Figure 3).

The exact mechanisms by which phospholipid scramblases regulate autophagy remains to be elucidated. Current evidence generally suggests a model that VMP1 and TMEM41B scramblases may act upstream of lipid transfer protein ATG2 and VPS13D to help equilibrate the concentrations of phospholipids on the outer and inner leaflets of the ER membrane during autophagosome expansion, allowing for continued inter-organelle phospholipid transport. While both VMP1 and TMEM41B may regulate autophagy at the same step by promoting the closure of autophagosomes, they may also have distinctive non-redundant roles as single deletion of either VMP1 or TMEM41B in cells is sufficient to block autophagic flux despite the presence of the other protein. However, overexpression of VMP1 can correct autophagy defect in TMEM41B-deficient cells but not vice versa ^7^, suggesting that VMP1 is either predominant or downstream of TMEM41B in regulating autophagy. Additionally, VMP1 and TMEM41B have been shown to regulate ER membrane structure ^14, 34^. It is conceivable that deficiency of VMP1 or TMEM41B may affect the fluidity and curvature of the ER and autophagosome membranes by exacerbating the imbalance in phospholipid content between the outer and inner membrane leaflet, resulting in stalled expansion of autophagosome membranes. Future lipidomic analysis using purified ER and autophagosome membranes for lipid profiles in wild-type and VMP1 or TMEM41B deficient cells may provide more definite insights into the mechanisms involved in scramblase regulation of autophagy and other organelle functions. Developing novel assays to directly measure scramblase activities in intact cells or tissues would also be helpful to advance our understanding of both molecular mechanisms and pathophysiology of scramblases.

## Scramblase Involvement in VLDL Secretion, Lipid Droplet Accumulation and NASH

Hepatic VLDL secretion is important for lipid homeostasis, and impaired VLDL secretion often leading to NAFLD ^35^. VLDL, a lipoprotein secreted from the liver, is responsible for transporting lipids, particularly triglycerides, through systemic circulation. In humans, all lipoproteins contain phosphatidylethanolamine (PE), phosphatidylinositol (PI), and sphingomyelin (SM), phosphatidylcholine (PC), and lyso-PC, with PE and SM being enriched in VLDL and LDL^36^. These phospholipids are synthesized on the cytoplasmic side of the ER membrane, necessitating the presence of scramblases to flip the newly created phospholipids luminally, both to equilibrate phospholipid content of the ER membrane and to provide material for VLDL assembly a process that begin in the ER and require apolipoprotein B100 (APOB100). Following assembly, the nascent lipid-poor VLDL is further matured to lipid-rich VLDL in the ER lumen and then exported and trafficked to the Golgi complex within coat protein complex II (COPII)-coated vesicles, where VLDL is further modified before eventually being secreted from hepatocytes ^37^. Recent work from Dr. Mizushima’s group elegantly demonstrated that VMP1 is critical for VLDL secretion ^15^. Using siRNA to silence *VMP1* in HepG2 cells, a human hepatoma cell line, they found that the resulting cells have increased accumulation of neutral lipids (triglyceride and cholesterol). Subsequent EM and immunofluorescence studies on the VMP1-depleted HepG2 cells revealed that neutral lipids were stalled inside the ER membrane bilayer resulting in severely impaired VLDL secretion. Defective VLDL secretion was also confirmed in intestinal epithelial cells and hepatocytes in *vmp1*-deficient zebrafish. Interestingly, deletion of either *rb1cc1/fip200* or *atg5*, two essential autophagy-related genes, did not affect VLDL secretion in zebrafish ^15^, suggesting autophagy may be dispensable for VLDL secretion, though the global KO resulting in animal lethality prevented a quantitative assessment of lipid homeostasis. A recent study from Wang and colleagues have reported that secretion-associated Ras-related GTPase 1B (SAR1B), a small GTPase that is critical for the formation of COPII vesicles, interacts directly with surfeit locus protein 4 (SURF4), a high efficiency cargo receptor ^38^. Hepatic deletion of either SAR1B or SURF4 in mice impairs hepatic VLDL secretion resulting in dramatic depletion of plasma lipids ^38^. Seeking to identify factors involved in regulating VLDL secretion that complex with hepatic SURF4, Huang et al. used a quantitative mass spectrometry approach to identify TMEM41B as a component in the hepatic FLAG-SURF4 immune-complex using an anti-flag resin ^14^. In subsequent studies employing confocal microscopy and immunoprecipitation (IP), the researchers found that TMEM41B interacts with both SURF4 and APOB, possibly forming a ternary complex necessary for lipid transport, which was not reflected in the closely related protein VMP1. Together with lipid depletion in circulation, an absence of mature lipoproteins and degradation of ApoB100 in the liver was also observed upon hepatic TMEM41B inactivation. ^14^ The data thus demonstrate a requirement of TMEM41B in lipoprotein biogenesis, consistent with its identity as a phospholipid shuttling scramblase.

Huang et al. then employed bulk liver RNA sequencing to analyze the molecular basis for TMEM41B knockout-induced dyslipidemia and fatty liver. They found that TMEM41B knockout resulted in an upregulation not only of factors involved in lipid peroxidation and the Land’s cycle, but also of those involved in lipid production, which was confirmed biochemically. Mass spectrometry showed a consistent accumulation of triglyceride, cholesterol, and diacylglycerol (DAG) along with a reduction of phospholipids. Huang et al. made note of this paradoxical activation of lipid synthesis despite the knockout cells already having been inundated with lipid droplets. This finding is shocking, accumulation of hepatic lipids due to secretion defect would normally be expected to result in feedback inhibition of de novo lipogenesis. To further understand this paradox, they investigated the sterol regulatory element binding protein (SREBP) cleavage-activating protein (SCAP)/SREBP pathway which revealed that loss of TMEM41B resulted in constitutional SCAP trafficking to the Golgi even in the presence of sterols, leading to increased activation of SREBPs. Increased SREBP activation is likely a secondary effect of TMEM41B loss as increased intracellular cholesterol is likely stalled in the ER membrane bilayer (although not directly assessed in this study) like in VMP1 deficient cells. Measurement of TMEM41B in mice with diet or genetically induced obesity revealed that TMEM41B levels were decreased compared to standard condition controls, indicating decreased TMEM41B may be a general mechanism for the development of NAFLD, upregulating de novo lipogenesis while also downregulating lipid secretion, and may serve as a potential therapeutic target for NAFLD.

Analysis of transmission electron microscopy images of hepatic ER revealed major structural differences between wild-type and TMEM41B knockout mice. In TMEM41B single knockout as well as TMEM41B/SURF4 double knockout mice, the ER was described as highly curved, sparse, and mildly dilated with a complete absence of lipoproteins within, the Golgi complex in these cells also lacking lipoproteins ^14^. The unique curved ER structure, which was found to encapsulate lipid droplets, was also confirmed through Focused Ion Beam – Scanning Electron Microscopy (FIB-SEM) imaging with High-Pressure Freeze (HPF). Hematoxylin and Eosin (H&E) staining revealed ballooning hepatocytes and positive Oil Red O staining revealed massive neutral lipid accumulation. Subsequent immunoblotting revealed a loss of APOB100 compared to APOB48 in TMEM41B knockouts, suggesting a deficiency in lipidation. While our group also found deformed and highly dilated ER in VMP1-deficient mouse pancreatic acinar cells, no obvious accumulated LDs were observed ^34^, suggesting that impaired neutral lipid secretion in VMP1- or TMEM41B-deficient cells may be cell type dependent.

Huang et al. have shown a possible role of TMEM41B in the development of steatohepatitis in addition to its role in autophagy. However, it is yet unknown whether TMEM41B’s role in autophagy and in lipid homeostasis are mediated by the same scramblase protein domain. Huang et al. put forth a model of scramblase deficiency causing morphological changes in the ER membrane as a possible novel stress response pathway, though the mechanism through which the cell recognizes and responds to excessive ER curvature needs much exploration. It would also be interesting to explore whether induction of ER curvature through an alternative pathway would be possible and would result in the same phenotype.

Although more than one scramblase protein exists in cells, in recent studies on scramblases, knockout of a single scramblase in hepatocytes results in full blown NASH, indicating that each of these proteins are not redundant in their function. Indeed, our group has found that hepatocyte-VMP1 knockout mice also had impaired VLDL secretion and developed NASH (manuscript accepted by Journal of Hepatology), suggesting the presence of TMEM41B is not sufficient to compensate for the loss of VMP1 and vice versa. Moreover, liposomal studies have shown that VMP1 and TMEM41B do not seem to require each other to have scramblase activity as TMEM41B can scramble phospholipids regardless of the presence of VMP1 ^14, 18, 19, 39^. Interestingly, overexpression of VMP1 was found to be able to correct the redistribution of plasma membrane cholesterol in TMEM41B deficient cells and *vice versa* ^18^. As discussed above, overexpression of VMP1 can rescue impaired autophagy in TMEM41B-deficient cells but overexpression of TMEM41B cannot rescue autophagy defects in VMP1-deficient cells ^16^. These data collectively suggest that VMP1 and TMEM41B functionally interact with each other and are incompletely redundant. It would be interesting to see whether overexpression of VMP1 or other scramblases would be able to correct TMEM41B dysfunction *in vivo* mouse models.

In a very recent publication, ATG9 was discovered to also be necessary in lipid mobilization, though only in human cell lines and *C. elegans*. Within human cell lines, knockout of ATG9A was observed to result in an increased size and number of lipid droplets ^4^. Specifically, ATG9A and ATG2A/B were shown via immunofluorescence to be necessary for fatty acid mobilization to mitochondria. Furthermore, ATG9A and TMEM41B structures were observed to interact through ATG2A/B, with ATG9A and TMEM41B being co-purified using tandem affinity purification ^4^. However, more research involving mammalian models are needed to determine if loss of ATG9 similarly results in dysfunctional lipid clearance.

## Future Perspectives

While TMEM41B knockout leading to secretory dysfunction is easily discernable from its critical function in mediating membrane formation, its ability to induce uncontrolled SREBP activation is unexpected and needs further research into the mechanisms involved. It is possible that SREBP activation may be related to a novel stress pathway induced by unmatched bilayers caused by a lack of scramblases. Future research may address whether TMEM41B function or ER morphology are more important regulators of SREBP activity, and whether VMP1 would have a similar role in SREBP regulation.

TMEM41B and VMP1 have been shown both to interact with nascent autophagosomes as well as to be involved in generation of lipoproteins. Consistent with this ability, TMEM41B has been found to accumulate at ER exit sites, forming a SURF4-TMEM41B-lipoprotein ternary complex, though it is yet unknown if this is reflected in VMP1. Further research is needed to determine whether TMEM41B, which has been shown to be involved in both ER-autophagosome and ER-LD contact, may be an integral protein at many or all ER-organelle contact sites, particularly ER-mitochondria contact sites, or if it is limited to the two. This would be especially important considering TMEM41B’s role in aiding in membrane and expansion. Further research into this field may unveil whether TMEM41B and VMP1 have similar or distinct roles in inter-organelle signaling.

Finally, impaired VLDL secretion and increased lipid synthesis may not be sufficient to explain the rapid development of NASH phenotypes in liver specific TMEM41B or VMP1 knockout mice. As discussed above, liver specific SURF4 knockout mice have impaired VLDL secretion with near zero plasma lipids, yet they only present with steatosis without further progression to NASH. While liver-specific ablation of *atg5*, an essential autophagy-related gene, neither affects hepatic VLDL secretion nor results in steatosis in mice ^40,41^, it does result in increased hepatocyte death, inflammation, liver fibrosis, and ultimately liver adenoma ^42–45^. Therefore, it is highly likely that the rapid development of NASH in TMEM41B or VMP1 knockout mice is due to combined defects on autophagy and lipid homeostasis.

Like VMP1 and TMEM41B, loss of ATG9A has been associated with accumulation of LDs within the cytoplasm of cells ^4^. Although this indicates that ATG9A is necessary for normal lipid homeostasis within cells, it is not yet known whether the mechanism by which this occurs is the same as that of VMP1 and TMEM41B. Conditional liver-specific knockout of ATG9 has not yet been explored, knockout of ATG9 having only been performed in a limited number of models, largely in vitro. Thus, it is unknown whether knockout of ATG9 within mouse liver would phenocopy TMEM41B and VMP1, resulting in rapidly progressive severe NASH. One important consideration when studying disease models generated by scramblase ablation may be the location of the protein within the cell and the cellular processes that are then disrupted.

In summary, while recent research on several scramblases has advanced our understanding of their roles in regulating autophagy, lipid homeostasis and perhaps viral infection, many unanswered questions remain. Why and how do different scramblases have distinct functions? How does depletion of these scramblases result in distinct phenotypes? Do the same scramblases in different cell types and tissues have different functions? What are other unknown functions of TMEM41B and VMP1 such as in regulation of mitochondria-ER contact and VLDL secretion? Are scramblases involved in all cellular processes involving only single leaflets of membrane bilayers? Are any human genetic mutations and polymorphisms of VMP1 or TMEM41B associated with NAFLD/NASH?.

## Abbreviations

ATG9: autophagy-related 9
COPII: coat protein complex II
ER: endoplasmic reticulum
HCC: hepatocellular carcinoma
MFN2: mitofusin 2
NAFLD: Non-alcoholic fatty liver disease
NASH: non-alcoholic steatohepatitis
SNX4: sorting nexin 4
SURF4: surfeit locus protein 4
TMEM41B: transmembrane protein 41B
T2DM: type 2 diabetes mellitus
VMP1: vacuole membrane protein 1 (VMP1)
VLDL: very-low-density lipoprotein
VPS13D: vacuolar protein sorting 13 homolog D
VTT: VMP1, TMEM41B and Tvp38
